# Ca^2+^/Calmodulin-Dependent Kinase Kinase α Is Expressed by Monocytic Cells and Regulates the Activation Profile

**DOI:** 10.1371/journal.pone.0001606

**Published:** 2008-02-13

**Authors:** Christopher B. Guest, Eric L. Deszo, Matthew E. Hartman, Jason M. York, Keith W. Kelley, Gregory G. Freund

**Affiliations:** 1 Division of Nutritional Sciences, University of Illinois at Urbana-Champaign, Urbana, Illinois, United States of America; 2 Department of Animal Sciences, University of Illinois at Urbana-Champaign, Urbana, Illinois, United States of America; 3 Department of Pathology, University of Illinois at Urbana-Champaign, Urbana, Illinois, United States of America; 4 Department of College of Medicine, University of Illinois at Urbana-Champaign, Urbana, Illinois, United States of America; University of California Merced, United States of America

## Abstract

Macrophages are capable of assuming numerous phenotypes in order to adapt to endogenous and exogenous challenges but many of the factors that regulate this process are still unknown. We report that Ca^2+^/calmodulin-dependent kinase kinase α (CaMKKα) is expressed in human monocytic cells and demonstrate that its inhibition blocks type-II monocytic cell activation and promotes classical activation. Affinity chromatography with paramagnetic beads isolated an approximately 50 kDa protein from nuclear lysates of U937 human monocytic cells activated with phorbol-12-myristate-13-acetate (PMA). This protein was identified as CaMKKα by mass spectrometry and Western analysis. The function of CaMKKα in monocyte activation was examined using the CaMKKα inhibitors (STO-609 and forskolin) and siRNA knockdown. Inhibition of CaMKKα, enhanced PMA-dependent CD86 expression and reduced CD11b expression. In addition, inhibition was associated with decreased translocation of CaMKKα to the nucleus. Finally, to further examine monocyte activation profiles, TNFα and IL-10 secretion were studied. CaMKKα inhibition attenuated PMA-dependent IL-10 production and enhanced TNFα production indicating a shift from type-II to classical monocyte activation. Taken together, these findings indicate an important new role for CaMKKα in the differentiation of monocytic cells.

## Introduction

Macrophages are capable of assuming numerous phenotypes depending on their microenvironment. Three broad categories of macrophage activation are-classical, type-II (innate) and alternative. Classical activation of macrophages results from exposure to IFNγ followed by TNFα stimulation [1]–[3]. Classically activated macrophages increase their surface expression of CD86 [3], [4] and produce TNFα, IL-12, oxide radicals, and chemokines [3], [5], [6]. The ligation of the Fc receptors for IgG along with stimulation of Toll-like receptors, CD40, or CD44 results in type-II activation of macrophages [3], [7]. Type-II activated macrophages show enhanced expression of CD86 [3] and generate the cytokines TNFα, IL-1, and IL-6 [7]. These macrophages, however, also elaborate IL-10, which differentiates them from classically activated macrophages [7], [8]. The third type of activation, alternative activation, fails to up-regulate CD86 [3], [9] but does enhance macrophage production of arginase [10], IL-1 receptor antagonist [11] and IL-10 [9]. Interestingly, the activation of this pathway results in macrophages with a reduced ability to kill microbes [12] . Therefore, classical activation appears to initiate the inflammatory process through production of the pro-inflammatory cytokines TNFα, IL-1 and IL-6. Type-II activation likely modulates and/or reduces inflammation by inducing Th2 helper T-cells [7], [8], [13] while increasing synthesis of the anti-inflammatory cytokine IL-10. Alternative activation directs macrophages to a repair phenotype [14]–[16].

Phorbol-12-myristate-13-acetate (PMA)-induced macrophage activation leads to increased expression of CD86 [17] indicating a classical or type-II activation phenotype. Importantly, studies employing PMA and calcium ionophores have linked IFNγ-dependent macrophage activation to pathways requiring both protein kinase C (PKC) and intracellular Ca^2+^ elevation [18]–[29]. Increased intracellular Ca^2+^ following PMA stimulation [27], [28] is important as both a co-factor for the conventional PKC isoforms activated by PMA [30] and the activation of the Ca^2+^/calmodulin (Ca^2+^/CaM) pathway through binding to CaM [31]. CaM interacts with a wide array of kinases and phosphatases [32], most notably the Ca^2+^/calmodulin-dependent kinase (CaMK) cascade. Interestingly, Ca^2+^/CaM interaction with both CaMKs and the upstream kinase CaMK kinase (CaMKK) is required for activation of this pathway [33]–[36]. In addition to having a CaM binding domain (CBD) in common, each member of the CaMK cascade has a catalytic domain adjacent to a regulatory region containing an autoinhibitory domain (AID) and the CBD [31]. Binding of Ca^2+^/CaM to the CBD results in a conformation change in the AID that allows for substrate binding to the kinase in question [31].

Two isoforms of CaMKK have been identified, CaMKKα and CaMKKβ [13], [37], both of which have been found in the cytoplasm [38] and cell nucleus [31], [39], [40]. Prospective sequence analysis demonstrates that CaMKKα has a nuclear localization sequence (a.a. 456–474). The mechanics, however, behind subcellular localization of the CaMKKs in monocytic cells has not been previously investigated. CaMKKα has been shown to phosphorylate CaMKI and CaMKIV [37], mediate Ca^2+^-dependent protection from apoptosis during serum withdrawal through phosphorylation and activation of Akt [41], [42] and directly interact with serum and glucocorticoid-inducible kinase 1 (SGK1) [41]. As a result of the activation of CaMKIV, CaMKKα indirectly leads to the activation of ERK-2, JNK-1 and p38 [31], [43], [44]. In addition, CaMKKα can cross-talk with the adenylate cyclase/cAMP pathway [45]–[47]. In fact, this is one method for inhibiting CaMKKα activity, where treatment with forskolin, an adenylate cyclase activator, results in PKA activation and subsequent phosphorylation of CaMKKα on serine 458, within the CBD, and threonine 108, potentially involved in autoinhibition of CaMKKα [46], [47]. In addition, a direct means of CaMKKα inhibition was developed by Tokumitsu et al. with the generation of STO-609 [48]. STO-609 is an extensively studied selective inhibitor of CaMKKs, with little effect on PKCs *in vitro* and *in vivo* [48], [49]. Finally, little is known of CaMKK's biologic role outside of the central nervous system. We have previously demonstrated that PKCδ translocation to the nucleus is associated with monocyte activation so we investigated the nuclear lysates of PMA activated U937 cells for proteins absent from nuclear lysates of non-PMA stimulated monocytes [17]. Here we demonstrate that human monocytic cells express CaMKKα, that expression of CD86, CD11b, TNFα, and IL-10 is regulated by CaMKKα and that inhibition of CaMKKα nuclear translocation is associated with blocking type-II monocytic cell activation and promoting classical activation.

## Results

### PMA induces CaMKKα nuclear localization

**Figure 1:**
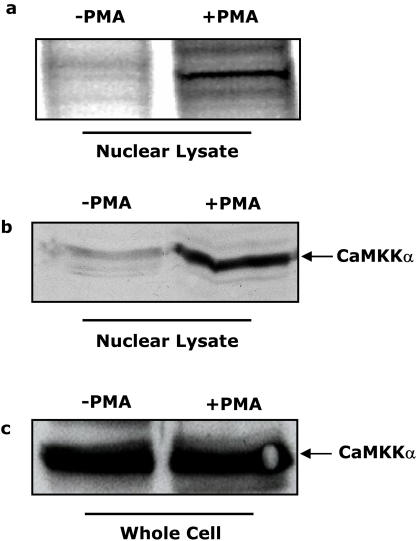
PMA induces CaMKKα nuclear localization. (A) U937 cells were treated with (+) or without (−) 100 nM PMA for 48 h and nuclear lysates were incubated with paramagnetic beads were as the solid phase. After extensive washing, proteins were resolved and visualized by SDS-PAGE and silver staining. Results are representative of three independent experiments. (B) U937 cells were treated and nuclear lysate affinity chromatography was performed as in panel A. CaMKKα mass was quantified by Western analysis. Results are representative of three independent experiments. (C) U937 cells were treated as in panel A. Affinity chromatography of whole cell lysates was performed as indicated. CaMKKα mass was quantified by Western analysis. Results are representative of three independent experiments.

Phorbol esters are potent stimulators of monocyte activation and have been used extensively to study macrophage differentiation [17]–[23], [26]–[29], [50]. In order to examine protein expression differences between nuclear lysates from PMA treated and non-PMA U937 cells, paramagnetic bead-assisted affinity chromatography was performed. Figure 1A shows that, when U937 cells were treated with PMA for 48 h, nuclear lysates from these cells contained an approximately 50 kDa protein that could be affinity isolated. Nuclear lysates from non-PMA treated cells did not contain this distinct band. To initiate identification of this protein, the ∼50 kDa silver stained band identified in Figure 1A was excised from the gel, trypsin-digested and mass mapped by MALDI-MS. Fragment analysis tentatively identified this protein as CaMKKα (GenBank accession number AF099105). Confirmation of this identification was performed by Western analysis using an anti-CaMKKα antibody (Figure 1B). Western analysis demonstrated that CaMKKα was recovered by affinity chromatography of nuclear lysates from PMA-treated cells while only traces were detected from nuclear lysates from non-PMA treated cells. Finally, Figure 1C demonstrates that CaMKKα was present in whole cell lysates of both PMA treated and untreated U937 cells. In addition, CaMKKβ was not detected (data not shown). Taken together, these results confirm that CaMKKα is present in monocytic cells and indicates that it localizes to the nucleus of PMA stimulated cells.

### Inhibition of PKC decreases PMA-dependent up-regulation of CD86/CD11b expression

**Figure 2:**
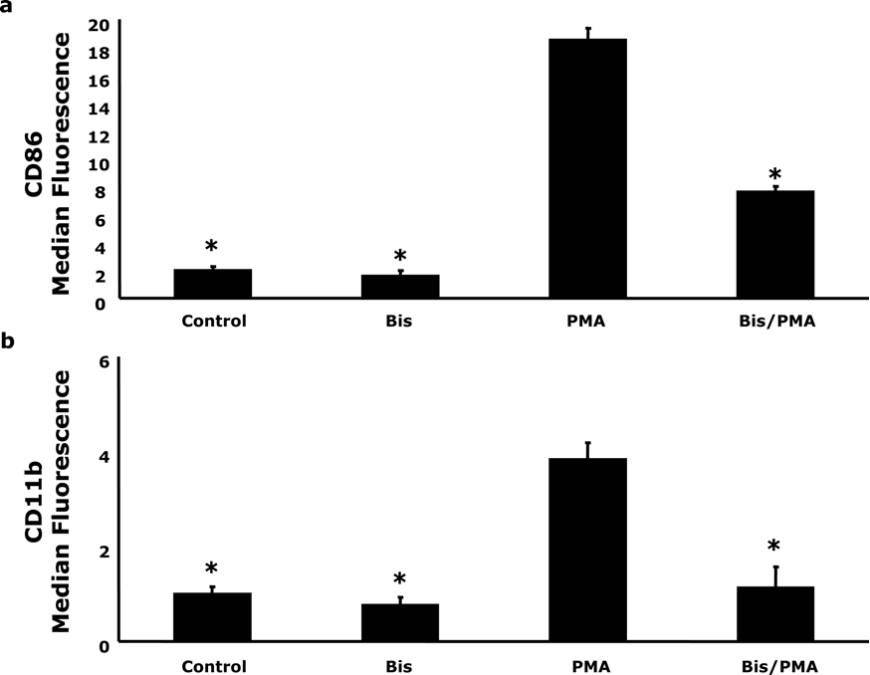
The PKC inhibitor Bisindolylmaleimide inhibits PMA-dependent upregulation of CD86/CD11b expression. (A & B) U937 cells were pretreated with or without 1 µM bisindolylmaleimide for 15 min prior to activation with 100 nM PMA for 48 h, as indicated. Surface expression of CD11b and CD86 was quantified by flow cytometry using FITC-conjugated CD11b and PE-conjugated CD86 antibodies. Results significantly different from PMA at α = 0.05 are indicated by (*). Results represent an average of three independent experiments±SEM.

To determine the role of PKC in the PMA-induced CD86/CD11b expression, bisindolylmaleimide inhibition studies were performed. Figure 2A–B show that when U937 cells were pretreated with bisindolylmaleimide for 15 min prior to PMA stimulation for 48 h cell surface CD86/11b expression was inhibited by more than 75% when compared to PMA treatment alone (P<0.05). This finding indicates that PKC plays a critical role in modulating PMA-induced activation of costimulatory markers.

### The CaMKKα inhibitor STO-609 enhances PMA-dependent up-regulation of CD86 but blocks CD11b expression and CaMKKα nuclear localization

**Figure 3:**
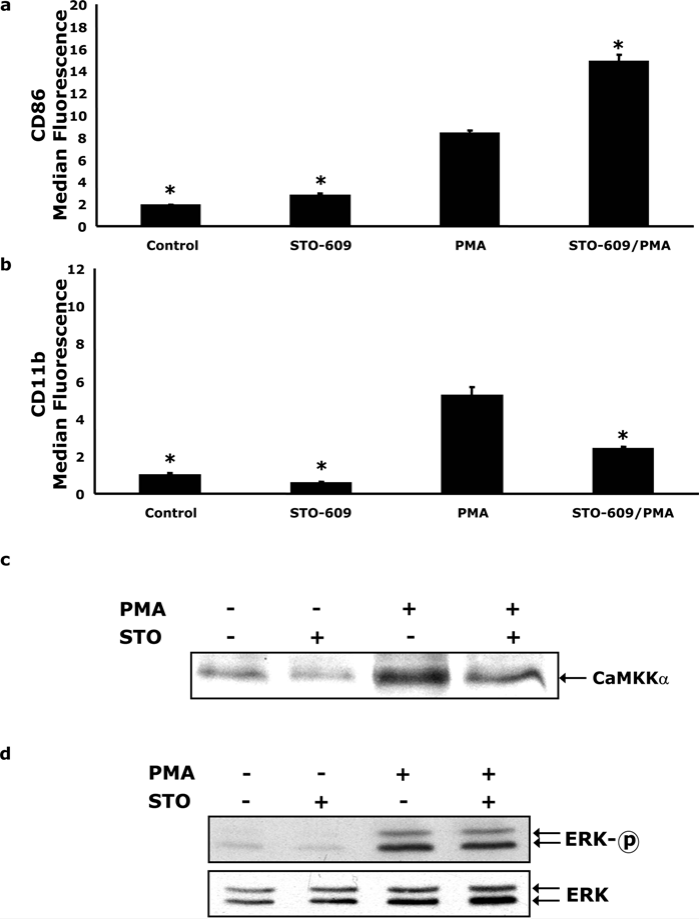
The CaMKKα inhibitor STO-609 enhances PMA-dependent up-regulation of CD86 but blocks CD11b expression and CaMKKα nuclear localization. (A and B) U937 cells were pretreated with or without 5 µg/ml STO-609 for 6 h prior to activation with 100 nM PMA for 48 h, as indicated. Surface expression of CD11b and CD86 was quantified by flow cytometry using FITC-conjugated CD11b and PE-conjugated CD86 antibodies. Results significantly different from PMA at α = 0.05 are indicated by (*). Results represent an average of three independent experiments±SEM. (C) Cells were treated as in panel A and CaMKKα was quantified by Western analysis of nuclear lysates. Results are representative of three independent experiments. (D) U937 cells were treated as in panel A and ERK1/2 phosphorylation (ERK-p) and mass (ERK) were measured by Western analysis in whole cell lysates. Results are representative of three independent experiments.

To determine the role of CaMKKα in PMA-dependent monocytic cell activation, STO-609 inhibition studies were performed. Figure 3A shows that, when U937 cells were pre-treated with STO-609 for 6 h prior to PMA addition for 48 h, cell surface CD86 expression was increased nearly 77% over PMA treatment alone (P<0.05). In contrast, STO-609 reduced PMA-dependent CD11b cell surface expression by 50% (P<0.05) (Figure 3B). Next, the consequence of STO-609 on PMA-dependent CaMKKα nuclear localization was examined. Western analysis (Figure 3C) demonstrated that STO-609 reduced CaMKKα nuclear localization by 50% (P<0.05). Similar to Figure 1C, PMA and/or STO-609 treatment had no impact on whole cell recovered CaMKKα (data not shown). Importantly, we have shown that ERK1/2 activation regulates CD86 and CD11b expression [17]. Interestingly, inhibition of CaMKKα with STO-609 had no impact on PMA-dependent activation of ERK1/2 (Figure 3D). These findings indicate that STO-609 inhibited PMA-induced up-regulation of CD11b and augmented PMA-stimulated CD86 expression. STO-609 also appears to inhibit PMA-dependent CaMKKα nuclear localization.

### Forskolin enhances PMA-dependent CD86 expression while inhibiting CD11b up-regulation and CaMKKα nuclear localization

**Figure 4:**
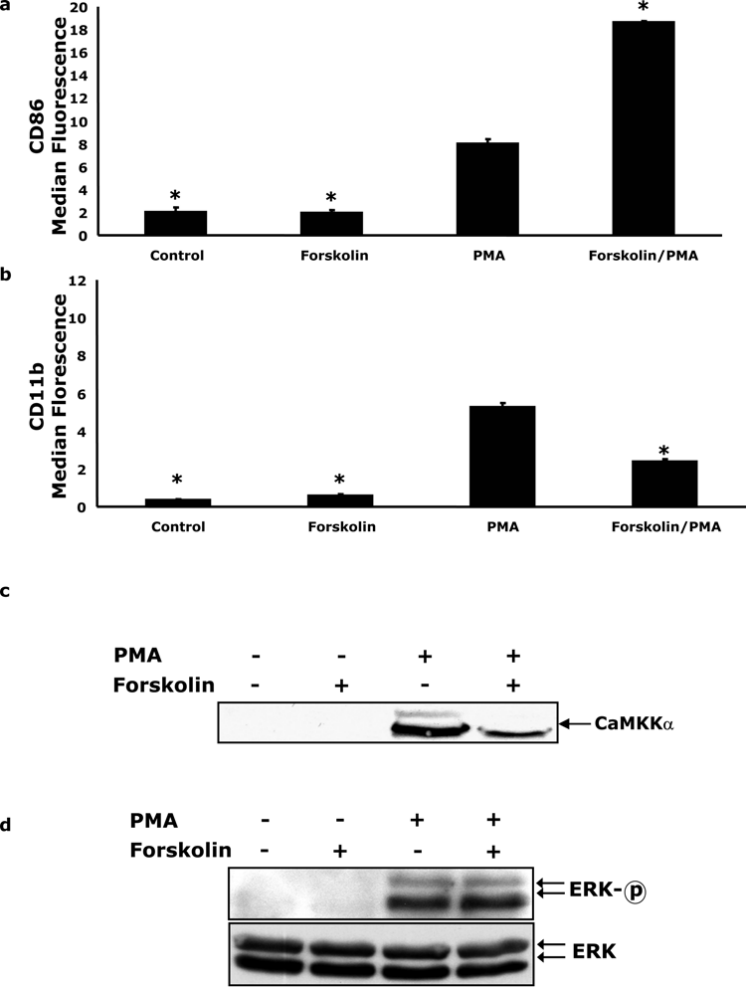
Forskolin enhances PMA-dependent CD86 expression while inhibiting CD11b up-regulation and CaMKKα nuclear localization. (A and B) U937 cells were pretreated with or without 30 µM forskolin for 1 h prior to activation with 100 nM PMA for 48 h, as indicated. Surface expression of CD11b and CD86 was quantified by flow cytometry using FITC-conjugated CD11b and PE-conjugated CD86 antibodies. Results significantly different from PMA at α = 0.05 are indicated by (*). Results represent an average of three independent experiments±SEM. (C) Cells were treated as in panel A and CaMKKα was quantified by Western analysis of nuclear lysates. Results are representative of three independent experiments. (D) U937 cells were treated as in panel A and ERK1/2 phosphorylation (ERK-p) and mass (ERK) were measured by Western analysis in whole cell lysates. Results are representative of three independent experiments.

Activation of adenylate cyclase leads to PKA-dependent phosphorylation and inhibition of CaMKKα [46], [47]. To determine the impact of PKA-mediated inhibition of CaMKKα on PMA-dependent up-regulation of CD86 and CD11b, activation studies were performed with the potent adenylate cyclase activator, forskolin [45]. Figure 4A shows that, when U937 cells were pre-treated with forskolin for 1 h prior to PMA addition for 48 h, cell surface CD86 expression was increased 130% over PMA treatment alone (P<0.05). Conversely, forskolin decreased PMA-dependent CD11b cell surface expression by 50% (P<0.05) (Figure 4B). PMA-dependent nuclear localization of CaMKKα was examined by Western analysis (Figure 4C) and was reduced by 60% after forskolin pretreatment (P<0.05). Similar to Figure 1C, PMA and/or forskolin treatment had no impact on whole cell recovered CaMKKα (data not shown). Figure 4D demonstrates that forskolin like STO-609 (Figure 3D) had no impact on ERK1/2 phosphorylation or mass. These findings indicate that forskolin inhibits PMA-induced up-regulation of CD11b and augments PMA-stimulated CD86 expression. Forskolin, also appeared to inhibit PMA-dependent CaMKKα nuclear localization.

### Inhibition of CaMKKα by siRNA enhances PMA-dependent up-regulation of CD86 but decreases CD11b expression

**Figure 5:**
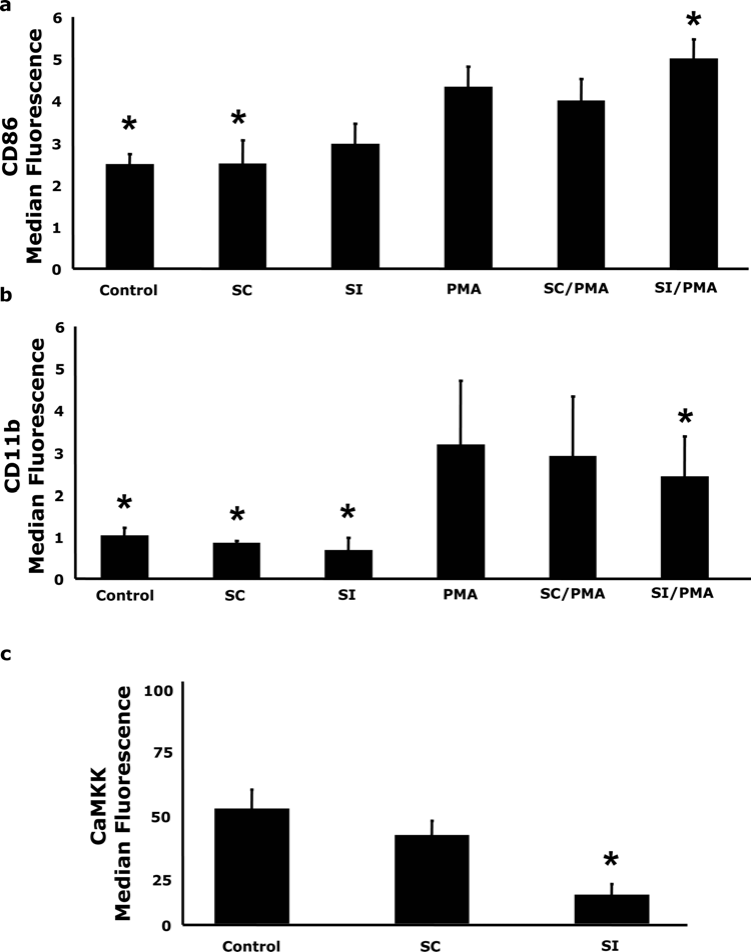
Inhibition of CaMKKα by siRNA enhances PMA-dependent up-regulation of CD86 but decreases CD11b expression. (A and B) Cells were treated with 0.25 µg of either CaMKKα siRNA or scramble siRNA control prior to stimulation with 100 nM PMA for 48 h. Surface expression of CD11b and CD86 was quantified by flow cytometry. Results significantly different from PMA at α = 0.05 are indicated by (*). Results represent an average of three independent experiments±SEM. (C) Cells were treated as in A and CaMKKα protein expression was quantified by intracellular flow. Results significantly different from control at α = 0.05 are indicated by (*). Results represent an average of three independent experiments±SEM.

Inhibition of CaMKKα with siRNA followed by treatment with PMA, enhanced CD86 expression by 17% (P<0.05) compared to PMA alone (Figure 5A) while PMA-induced CD11b expression was reduced by 24% (P<0.05) compared to PMA alone (Figure 5B). CaMKKα knockdown did not significantly affect PMA independent CD86 expression or CD11b. The siRNA scrambled control sequence did not significantly alter CD86 or CD11b expression in any of the conditions tested. Figure 5C indicates that CaMKKα expression was decreased by nearly 76% by treatment with CaMKKα siRNA (P<0.05) while the siRNA scramble control did not significantly affect CaMKKα expression. Taken together, these data indicate that knockdown of CaMKKα expression alters PMA-induced macrophage differentiation.

### CaMKKα inhibition enhances PMA-induced production of TNFα while reducing that of IL-10

**Figure 6:**
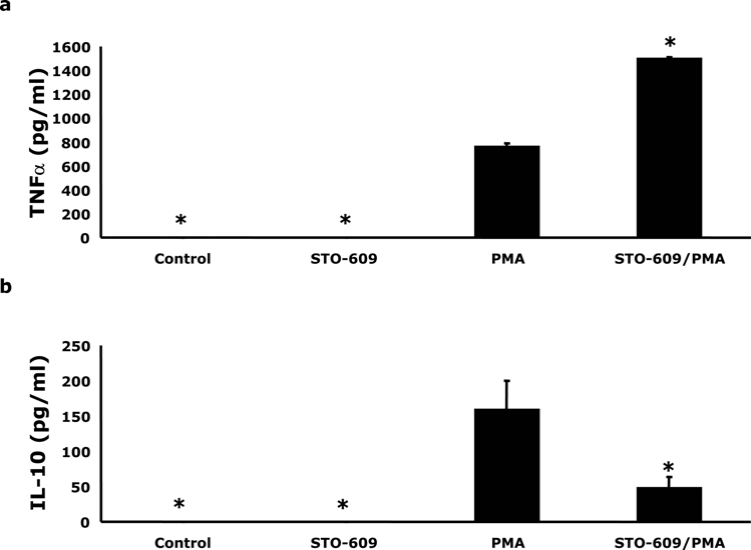
CaMKKα inhibition enhances production of TNFα while reducing that of IL-10. (A and B) U937 cells (1×10^6^ cells/treatment) were pretreated with or without 5 µg/ml STO-609 for 6 h prior to activation with 100 nM PMA for 48 h, as indicated. TNFα and IL-10 concentrations were quantified from culture supernatants by cytokine assays. Results significantly different from PMA at α  = 0.05 are indicated by (*). Results represent an average of three independent experiments±SEM.

Type-II activated macrophages produce both TNFα and IL-10 while classically activated macrophages generate just TNFα [3]. To determine if CaMKKα inhibition was important to macrophage cytokine production, cytokine assays were performed. Figure 6A demonstrates that PMA induced an increase in U937 cell TNFα production from 0 pg/ml to 764±22 pg/ml. STO-609 pre-treatment enhanced this response by 97% (P<0.05) while having no impact on basal TNFα production. U937 cell IL-10 elaboration was also increased by PMA from 0 pg/ml to 160±40 pg/ml (Figure 6B). In contrast to TNFα, STO-609 pre-treatment reduced IL-10 production by 70% (P<0.05). Production of the inflammatory cytokines IFN-γ and IL-2 or the anti-inflammatory cytokines IL-5 and IL-4 were not detected (data not shown). Taken together, these data show that inhibition of CaMKKα reduces the production of IL-10 while enhancing that of TNFα indicating a shift from type-II to classical monocyte activation.

## Discussion

The co-stimulatory molecule, CD86, is expressed on both classical and type-II activated macrophages and is necessary to define a classically activated macrophage [3]. We [17] and others [51] have previously shown that during monocytic cell activation CD86 up-regulation requires ERK1/2. Up-regulation, however, of CD86 during type-II activation has not been thoroughly studied. Figure 1A shows that a prominent ∼50 kDa band was affinity purified from nuclear lysates derived from PMA-activated U937 cells. Combining MALDI-MS fragment analysis with subsequent Western analysis using a CaMKKα specific antibody, a component of this band was identified as CaMKKα (Figure 1B). Next, CaMKKα was isolated from whole cell lysates of both PMA treated and untreated U937 cells. Importantly, PMA did not increase CaMKKα mass in whole cells (Figure 1C) or nuclear lysates from non-PMA treated cells (Figure 1B). Taken together, these data indicate that PMA-dependent activation of monocytic cells is associated CaMKKα nuclear translocation. Importantly, the identification of this enzyme in the U937 cell line may provide new avenues for the treatment of monocytic cell leukemia. Indeed, the potential of CaMKKs as therapeutic targets is currently being investigated by our laboratory.

Given the central role of monocytes in the immune response and the importance of coordinating activation in specialized microenvironments [52] we investigated the effect of PMA stimulation on CD86/CD11b expression. We have previously shown that PKCδ plays a critical role in IL-4 mediated PMA-induced CD86/CD11b expression [17], however, prolonged exposure to PMA has been shown to down-regulate certain PKCs [53] so we preformed experiments to investigate the effects of PKC inhibition. Treatment with bisindolylmaleimide significantly attenuated PMA-induced CD86 and CD11b expression (Figure 2A–B) indicating that activation of PKCs was essential to PMA-induced CD86 and CD11b expression. Next, in order to determine if CaMKKα regulated CD86 expression, CD86 expression was examined in PMA-activated U937 cells. Inhibition of CaMKKα with STO-609 [48] increased PMA-dependent CD86 expression nearly 77% while PMA-induced CD11b expression was attenuated by 50% (Figures 3A–B). STO-609 also impaired the translocation of CaMKKα into the nucleus following PMA-activation (Figure 3C). This is consistent with the hypothesis that activation of CaMKKα may lead to nuclear targeting of this protein and initiation of downstream events [38]–[40]. ERK1/2 is critical to activation-dependent monocytic cell CD86 expression. We have shown that CD86/CD11b expression after activation requires ERK1/2 [17]. Notably, CaMKKα can lead to the activation of ERK-2 [44]. To determine if CaMMKα regulated ERK1/2, STO-609 inhibition of CaMMKα was utilized. As Figure 3D shows, PMA-dependent ERK1/2 activation was not enhanced or decreased by STO-609. While the PKC and Ca^2+^/CaM pathways can interact [30], [54]–[56], our data may represent that under strong MAPK stimulation, via PMA-PKC, the phosphorylation of ERK by CaMK is redundant and consequently inhibition of CaMKK is without effect.

Work by Wayman et al. and Matsushita et al. demonstrated that forskolin inhibits CaMKKα activity by inducing PKA-dependent [45] phosphorylation of serine 458, within the CaMKKα calmodulin binding domain, and threonine 108, involved in autoinhibition of CaMKKα [46], [47]. To investigate the impact of a second mechanism of CaMKKα inhibition on PMA-induced CD86/CD11b expression, we used forskolin, a potent activator of adenylate cyclase [45]. Figures 4A–B demonstrates that forskolin, itself, does not impact CD86 or CD11b expression. This result was expected because we have shown that PMA dependent-up-regulation of CD86 requires ERK1/2 activation [17] and Figure 4D demonstrates that forskolin alone did not activate ERK1/2. However, when forskolin was used in conjunction with PMA, a doubling of CD86 expression and an attenuation of CD11b up-regulation was observed. Additionally, CaMKKα nuclear translocation was blocked by forskolin (Figure 4C), while PMA-stimulated ERK-1/2 activation was unaffected (Figure 4D). These effects mimicked that of STO-609 (Figure 3), an inhibitor of CaMKKα that works through an entirely different mechanism [48]. Although the use of two pharmacological inhibitors of CaMKKα that act through different mechanisms provides strong evidence that CaMKKα regulates PMA-induced CD86 and CD11b expression, direct molecular interventions provide the strongest evidence. When CaMKKα expression was reduced by siRNA knockdown, PMA-dependent CD86 expression was enhanced and CD11b expression decreased as expected. Taken together, these findings indicate that CaMKKα is an important regulator of macrophage differentiation.

PMA activates both Ca^2+^-dependent and -independent events within the cell. Specific surface proteins on murine macrophages such as HLA-DR and FcγR are increased in response to PMA, which is dependent on increased Ca^2+^, but is not affected by the Ca^2+^/CaM or PKC pathways [19]. Cellular spreading of murine macrophages in response to PMA requires intracellular Ca^2+^ [28], treatment of malignant myeloid progenitor cells from patients with chronic myelogenous leukemia with a calcium ionophore leads to expression of CD86 and other surface markers including those for dendritic cell-activation and adhesion [57], and expression of CD86 in human monocyte-derived dendritic cells is also increased by elevated Ca^2+^ flux [58]. However, increased Ca^2+^ is not always the critical factor in PMA-mediated effects. In the case of macrophage priming, increased intracellular Ca^2+^ alone is not sufficient and stimulation with activators of PKCs, such as PMA, is needed to achieve priming of these cells [21], [22]. Arachadonic acid release and IgG-mediated phagocytosis following PMA activation of human monocytes occurs through activating a Ca^2+^-independent phospholipase A2 [59], potentially mediated by PKC. In addition, PMA has defined Ca^2+^-independent effects in glomerular mesangial cells [60], T lymphocytes [61], and mouse oocytes [62]. Therefore, we examined the impact of ionomycin, a Ca^2+^-ionophore, on PMA-induced CD86 expression and found no effect of ionomycin alone or in combination with PMA (data not shown). This indicates that increased CD86 expression requires more than increased Ca^2+^ and activation of the Ca^2+^/CaM pathway [63]. We have also shown that inhibition of PMA-dependent activation of PKCδ markedly augments CD86 expression while blocking CD11b expression in monocytic cells [17]. This effect was correlated with loss of PKCδ activation and failure of PKCδ to translocate to the nucleus. PKCδ is classified as a novel isoform in that it does not require Ca^2+^ but is dependent on diacylglycerol and phosphatidyserine for activity [64]. Therefore, one possibility to account for CaMKKα nuclear translocation is via an association with PKCδ. Since activation of CaMKKα appears to be important for nuclear targeting (Figures 3–4) interaction with PKCδ could represent a critical step in this regulatory process, although further evidence is needed to establish such a relationship.

Finally, an important distinction between classically activated macrophages and type-II activated macrophages is the type of cytokines produced. Both forms of activation result in the production of TNFα, however IL-10 is only elaborated during type-II activation [3]. IL-10 is a potent anti-inflammatory cytokine [65], [66] and Gerber et al. demonstrated that IL-10 was necessary for the ability of type-II activated macrophages to rescue mice from a lethal dose of LPS [7]. Therefore, we examined the production of these two cytokines following monocytic cell activation and found that inhibition of CaMKKα caused a 97% increase in TNFα production and a 70% decrease in IL-10 production (Figures 6A–B). Taken together these results indicate that PMA induces a type-II activation profile and that CaMKKα inhibition shifts the activation state to the classical pathway. Importantly, our data demonstrate a new role for CaMKKα in macrophage differentiation, and indicate an association between the inhibition of CaMKKα and its nuclear translocation.

## Materials and Methods

### Materials

The monocytic cell line U937 was purchased from American Type Culture Collection (Manassas, VA). SuperSignal® West Pico chemiluminescent substrate (cat. #34077) was purchased from Pierce (Rockford, IL). NitroBind®, Pure nitrocellullose membrane 0.45 µm (cat. #EP4HYB0010) was purchased from Osmotics (Westborough, MA). Fluorescein isothiocyanate (FITC)-conjugated anti-CD86 (cat. #MHCD8601-4), and R-Phycoerythrin (PE-R)-conjugated anti-CD11b (cat. #MHCD11b04) were purchased from Caltag (Burlingame, CA). Anti-phospho-ERK1/2- Thr202/Tyr204 (cat. #9106) was purchased from Cell Signaling Technology (Beverly, MA). Anti-CaMKKα(sc-11370) , anti-rabbit IgG-FITC antibody (sc-2012), CaMKKα siRNA (sc-29904), Control siRNA (sc-37007) and siRNA transfection reagent (sc-29528) was purchased from Santa Cruz Biotechnology (Santa Cruz, California). Anti-ERK1/2 (cat. #06182) was purchased from Upstate Biotechnology, Inc. (Lake Placid, NY). Forskolin (cat. #344270), and Bisindolylmaleimide (cat. #203293) were purchased from Calbiochem (La Jolla, CA). STO-609 (cat. #1551) was purchased from Tocris Cookson, Inc. (Ellisville, MO). All other cell culture reagents and chemicals were purchased from Sigma (Saint Louis, MO).

### Cell Culture

U937 cells were cultured in growth media (RPMI 1640 supplemented with 10% fetal bovine serum, 2.0 g/L sodium bicarbonate, 2.5g/L glucose, 100,000 units/L penicillin, and 100 mg/L streptomycin, 1 mM sodium pyruvate and 10 mM HEPES, pH 7.4) as previously described [17]. Cells were passaged 1:2 with fresh medium every three days. For all experiments, cells were washed twice and re-suspended in growth media at 1×10^6^ cells/ml with the indicated treatments.

### CaMKKα Knockdown

siRNA knockdown of CaMKK was performed according to the manufacturer's instructions. In brief, cells were cultured in antibiotic free media for 24 h washed twice and resuspended in 200 µl transfection media containing 0.25 µg of siRNA duplex (scrambled control or CaMKKα cocktail) and 2 µl of siRNA transfection reagent. Cells were incubated as described above for 6 h, then 500 µl of fresh transfection media was added and cells incubated for an additional 24 h. Finally, cells were washed and resuspended in growth media and treated with PMA for 48 h and assayed by flow cytometry. CaMKKα expression was quantified by intracellular flow cytometry.

### Nuclear Lysates

were isolated as previously described [17]. 1×10^6^ cells/test were stimulated as indicated and then washed with ice-cold DPBS, spun and resuspended in Buffer A (10 mM KCl, 1.5 mM MgCl_2_, 1 mM dithiothreitol (DTT), 1 µg/ml leupeptin, 1 µg/ml aprotinin, 1 mM PMSF and 10 mM HEPES, pH 7.9). After 15 min on ice, lysates were passed through a 26½ gauge needle three times and then centrifuged at 800×g for 10 min at 4°C. The nuclear pellet was re-suspended in Buffer A and layered over a 30% sucrose cushion (w/v in Buffer A) and centrifuged 15 min at 5000×g at 4°C. The purified nuclear pellet was then incubated in 25% glycerol, 400 mM NaCl, 10 mM KCl, 1 mM EDTA, 1 mM EGTA, 1 mM DTT, 1 mM PMSF, 1 µg/ml leupeptin, 1 µg/ml aprotinin and 10 mM HEPES, pH 7.9 with vortexing for 30 min at 4°C. After centrifugation at 16,000×g for 30 min at 4°C, the supernatant (nuclear extract) was analyzed

### Whole cell Lysates

were prepared as previously described [17]. In brief, 1×10^6^ cells/test were lysed in 1 ml of ice-cold lysis buffer (1% Triton X-100, 150 mM NaCl, 1 mM NaF, 1 mM EGTA, 1 mM EDTA, 1 mM phenylmethylsulfonyl fluoride (PMSF), 2 µg/ml aprotinin, 2 µg/ml leupeptin, 1 mM sodium orthovanadate and 50 mM Tris-base, pH 7.4) by double passage through a 26½-gauge needle. Lysates were cleared by centrifugation at 16,000×g for 10 min.

### Paramagnetic Bead Affinity Chromatography

U937 cell nuclear or whole cell lysates, generated as above (*Cellular Fractionation*), were diluted to 1 µg/ml with Buffer A (without Triton X-100) to ensure adequate column absorption and flow. Lysates and paramagnetic beads (Miltyni, Auburn, CA) were then incubated together at 21°C for 5min. Bead complexes were washed extensively with 10 mM KCl, 7% glycerol, 1 mM DTT, 10 mM HEPES, pH 7.9. Columns were then eluted with 1% SDS, 1 mM DTT, 10 mM Tris-HCl, pH 6.8, and proteins separated on 10% polyacrylamide gels. Gels were either electro-transfered to nitrocellulose for Western analysis or silver stained using the SilverQuest™ kit (Invitrogen, Carlsbad, CA). For silver staining, 15×10^6^ cells/test were used.

### Mass Spectrometry (MS)

Proteins were separated by SDS-PAGE and silver stained using SilverQuest (Invitrogen) following manufactures instructions. Silver stained bands were excised, de-stained (following manufactures instructions) and speed vacuum-dried until tacky. Gel pieces were rehydrated in 100 mM ammonium bicarbonate with 2 µg/ml trypsin and incubated at 37°C for 36 h. Trypsin digestion was stopped by addition of 10% trifluoroacetic acid (TCA) and peptides extracted into 50 µl of 10% TCA/60% acetonitrile. Samples were then concentrated by 1/3 via speed vacuum drying and further concentrated and desalted via strong cation exchange using ZipTip (Millipore) packed with SCX resin (Millipore). Peptide molecular ions were analyzed in the positive ion mode using a Voyager 4066 Mass Spectrometer (Applied Biosystems) where acceleration voltage was set at 20 kV and 100 laser shots were summed. To calibrate peptide digestion, equine myoglobin was used as an external standard. PeptIdent (Swiss Institute of Bioinformatics) was used to identify the monoisotope peptide masses where 1 missed cleavage was allowed and a minimum of 4 matching peptides was required for a match. The pI range was ±2.00 and the molecular weight range was ±20%.

### Flow Cytometry

was performed as previously described [50]. In brief, after indicated treatments, cells were incubated in growth media supplemented with 5 mM EDTA for 1 hr at 37°C and then washed once in 0.5% Dulbecco's phosphate-buffered saline (DPBS) containing 0.5% BSA (DPBS-BSA) without calcium and magnesium. Fluorochrome-conjugated antibodies at 10 µg/ml/test were added to 1×10^6^ cells and incubated on ice for 15 min and then washed with DPBS-BSA 0.5% Fluorescence was detected on an Epics XL flow cytometer (Beckman Coulter, Fullerton, CA) quantifying 1.5×10^4^ events using gates to exclude nonviable cells as determined by propidium iodide staining. For intracellular staining, cells were fixed in 2% formalin/PBS for 10 m at room temperature, washed twice with DPBS-BSA 0.5% and blocked with PBS containing 10% fetal bovine serum for 20 m. 100 µl of PBS/FBS containing 0.2 % triton and 10 ug/ml/test of CaMKKα antibody or isotype control was added and cells were allowed to incubate overnight at 4°C. Cells were then washed twice with DPBS-BSA 0.5% and blocked with PBS containing 10% fetal bovine serum for 20 m. 100 µl of PBS/FBS containing 0.2 % triton and 10 ug/ml/test anti-IgG-FITC detection antibody was added cells were allowed to incubate for 1 h at room temperature then washed twice with DPBS-BSA 0.5%. Fluorescence was detected as above. Cell viability for all experiments was at least 85%. Median values of each population were used to indicate the levels of expression of each antigen assayed.

### Western analysis

was performed as previously described [50]. Proteins were resolved by SDS-PAGE (1×10^6^ cells/lane) in 10% gels and then electro-transferred to nitrocellulose. Immunoreactive proteins were visualized with the indicated primary antibodies using enhanced chemiluminescence (ECL) (Amersham) followed by densitometry.

### Cytokine Assays

Measurement of IL-10, IL-4, TNF-α, IL-2, IL-5 and IFN-γ were performed using the Cytometric Bead Array Kit (BD PharMingen) as per the manufacturer's instructions. Fluorescence was detected on a Cytomation MoFlo flow cytometer (DakoCytomation Colorado Inc, Fort Collins, Colorado) quantifying 5×10^4^ events. Absolute cytokine amounts were calculated by comparison to a standard curve generated using a 3^rd^ order polynomial regression curve-fitting algorithm. Limits for detection were IL-10 (2.8 pg/ml), IL-4 (2.6 pg/ml) TNF-α (2.8 pg/ml), IL-2 (2.6 pg/ml), IL-5 (2.4 pg/ml) and IFN-γ (7.1 pg/ml).

### Statistical Analysis

Where indicated, experimental data was analyzed by the Student's t-test for comparison of medians using Microsoft Excel (Redmond, WA).
